# EASE-6G: An Energy-Aware SDN Framework with Proactive Slicing and DL-Based Overhead Mitigation for Scalable IoT Networks

**DOI:** 10.3390/s26123858

**Published:** 2026-06-17

**Authors:** Marwah Albeladi, Kamal Jambi, Fathy E. Eassa, Maher Khemakhem

**Affiliations:** Department of Computer Science, Faculty of Computing and Information Technology, King Abdulaziz University (KAU), Jeddah 21589, Saudi Arabia; fathy55@yahoo.com (F.E.E.); makhemakhem@kau.edu.sa (M.K.)

**Keywords:** 6G networks, energy efficiency, DL-based overhead mitigation, network slicing, DQN, LSTM, proactive flow management, SDN orchestration, deep learning

## Abstract

Sixth-generation (6G) networks are expected to enable a new level of connectivity, with peak data rates reaching 1 Tbps and latencies below 0.1 ms, especially in large-scale Internet of Things (IoT) environments. Despite these advantages, the rapid increase in device density poses multiple challenges, most notably the growth in control plane signaling and the associated increase in energy consumption. These issues might significantly affect the scalability and efficiency of future networks if left unaddressed. We propose EASE-6G, an energy-aware Software-Defined Networking (SDN) framework that moves network operation from reactive to proactive and predictive, supporting ultra-dense conditions, where the number of connected devices may reach $10^{6}$ devices per square kilometer. EASE-6G uses Proactive Flow Installation to reduce the need for instant decisions. Traffic is predicted using a Long Short-Term Memory (LSTM) model, while a signaling-aware Deep Q-Network (DQN) streamlines control, reducing unnecessary signaling while maintaining performance. Simulations in OMNeT++/Simu5G were performed to compare EASE-6G with Smart Fog Radio Access Network (SF-RAN) and Deep Q-Network-based Open Radio Access Network (DQN-ORAN). EASE-6G was found to reduce energy consumption by 36.8%, signaling overhead by 36.7%, and latency by 35.6%. The LSTM model achieved a Mean Absolute Percentage Error (MAPE) of 4.2%. The DQN agent showed improved stability, with 22% lower variance than the baseline. These results demonstrate that the proposed predictive SDN control mechanisms improve energy efficiency and reduce overhead, delivering a practical solution for the implementation of scalable, sustainable IoT in future 6G networks.

## 1. Introduction

In the context of the Internet of Things (IoT), sixth-generation (6G) network integration is expected to provide pervasive, wide-area connectivity, sub-millisecond air-interface latency, and terahertz-band ultra-wideband coverage. These architectures are anticipated to deliver peak data rates exceeding 1Tbps alongside user-experienced latencies below 0.1ms [1]. With the achievement of these performance targets, a new class of applications [2], from remote robotic surgery to closed-loop industrial automation and holographic mobility [3], may be supported.

However, the structural foundation of 6G remains a topic of active debate [4]. A primary point of friction is how future networks will support ultra-dense IoT deployments, particularly in massive Machine-Type Communications (mMTC) scenarios. With anticipated device densities easily exceeding $10^{6}$ nodes per ${\mathrm{km}}^{2}$ [5], energy sustainability becomes a paramount design constraint, as millions of battery-powered devices must maintain long operational lifecycles. Recent studies emphasize that addressing these challenges requires the explicit integration of energy consumption models directly into the system design and resource evaluation process [6]. Without such frameworks, standard control processes present vulnerabilities under the sheer volume of signaling traffic and its disproportionate energy demands [7,8].

Software-Defined Networking (SDN) is a key enabler of scalability in 6G networks [9], with SDN and Network Function Virtualization (NFV) recently emerging as central mechanisms for service delivery in next-generation architectures [10]. Through network slicing, SDN facilitates logical resource separation to manage competing priorities, handling the high reliability of Ultra-Reliable Low-Latency Communication (URLLC) alongside the bandwidth demands of Enhanced Mobile Broadband (eMBB). Despite this flexibility, the underlying reactive mechanics of traditional SDN configurations often undermine strict performance guarantees. The primary limitation lies in the basic Packet-In/Flow-Mod cycle.

Controllers typically intervene only after detecting a packet that lacks a registered route [11], and maintaining this reactive dependency actively drives network congestion. This reactive cycle triggers energy inefficiency due to repetitive message exchanges, which can consume nearly 25% of the active power budget under mMTC registration storms [12]. Consequently, the system risks signaling saturation under control plane loads exceeding 850 msg/s [13], and triggers deterministic latency, which may cause latency violations when flow initialization delays fall behind the rigid 1 ms threshold required for mission-critical tasks, such as real-time tactile internet control and autonomous robotic synchronization [14].

Prior research has explored various strategies for optimizing energy efficiency in 6G-IoT environments. Specifically, Albeladi et al. [15] examined the individual impacts of SDN dynamic slicing, duty cycling, and AI-driven slicing using hybrid Convolutional Neural Networks (CNNs) and Bidirectional Long Short-Term Memory (BiLSTM) models compared to a static non-slicing baseline. Their results demonstrated significant power reductions, including a 66.28% decrease (from 4.5 W to 1.51 W) through SDN slicing and a 60.14% reduction (from 3.8 W to 1.51 W) via CNN-BiLSTM classification, utilizing explained attribution techniques to validate model decision paths. While these strategies provide foundational insights into isolated network performance, EASE-6G builds upon this work by unifying these mechanisms into an integrated ecosystem that structurally links the volume of control plane signaling directly to overall energy limits.

Therefore, to resolve the performance gap caused by uncoordinated reactive control signaling and static resource slices under high traffic densities, we present EASE-6G. This ecosystem treats orchestration as a signaling-aware optimization problem, rather than a segmented management cycle. The specific analytical contributions of this study are as follows:

- Predictive Flow Pre-Installation: We engineered an LSTM-based forecasting model that isolates traffic dynamics ahead of packet arrivals. Yielding a 36.7% attenuation in signaling overhead through pre-emptive rule configuration, this strategy consistently outperformed Gated Recurrent Unit (GRU) and AutoRegressive Integrated Moving Average (ARIMA) alternatives in operational accuracy.
- Autonomous Signaling-Aware Orchestration: We developed a reinforcement learning agent using a Deep Q-Network (DQN) representing the value-based branch of deep reinforcement learning that dictates the distribution of resources based on signaling penalties. This approach produced a 29.9% improvement in resource allocation efficiency and achieved state convergence with 22% less variance when compared with standard O-RAN DQN (DQN-ORAN) baseline frameworks.
- Distributed Multi-Tier Sustainability: By adopting a hierarchical topology, edge-level request processing accounts for the signaling cost (γ), allowing the system to achieve total control-plane energy savings of 36.8% compared to a traditional reactive SDN framework.
- Enhancing Systemic Quality Attributes: Focusing on stability, scalability, and Quality of Service (QoS), we performed rigorous empirical testing utilizing OMNeT++. Under stress tests involving saturation loads and 15% device churn, EASE-6G recorded a 35.6% improvement in end-to-end latency compared to the reactive SDN baseline while enhancing critical systemic quality attributes.

The remainder of this paper is structured as follows. Section 2 summarizes existing architectures for deep-learning-based SDN optimization. Section 3 details the architectural design and experimental framework of the EASE-6G ecosystem. Section 4 discusses the empirical outputs and comparative results. Section 5 provides a comprehensive discussion of computational complexity, limitations, and training feasibility. Finally, Section 6 presents the conclusions and directions for future work.

## 2. Related Work

The ongoing transition toward 6G has naturally sparked interest in SDN-based orchestration logic. Despite this, existing deployments consistently overlook the sustainability aspects of control plane energy use. This section traces the relevant architectural history across decentralized control plane partitioning, AI-driven networking, and signaling load management.

- Distributed Edge Offloading and Processing: Shifting control mechanisms away from central servers represents a common tactic to manage SDN saturation. Zhang et al. [8] constructed a Smart Fog Radio Access Network (SF-RAN), actively offloading 70% of flow traffic directly to fog nodes to restrict backhaul interference. Although it successfully limits the data volume in static grids, SF-RAN fails to adapt under highly stochastic traffic spikes. More critically, it ignores the energy drain associated with inter-tier signaling entirely. Similarly, the Network Intelligence Stratum (NIS) designed by Soto et al. [16] curbs the control-to-data flow volume by deploying localized decision-making engines at the edge of the core network to filter redundant registration events. However, the subsequent coordination intervals, which average between 50 and 80 ms, render it functionally invalid for strict URLLC deployments.
- AI-Enhanced Resource Orchestration: Machine learning now heavily dictates baseline resource allocations in next-generation virtualized infrastructures [17]. Firouzi et al. [12] integrated DQN logic to specify Virtual Network Function (VNF) positioning, measuring a 35% decrease in service delay. However, the model remains intrinsically reactive, initiating logic shifts only after receiving congestion alerts. This inherent lag invites transient QoS failures, a latency gap that our system specifically bridges using proactive Long Short-Term Memory (LSTM) forecasting. While Mahamod et al. [13] successfully targeted mobility-based signaling density constraints, the localized scope side-stepped the massive Packet-In demands intrinsic to fixed IoT connections in urban environments.
- Energy-Aware Control Plane Management: Scientific scrutiny regarding the net energy footprint of AI-native network structures continues to rise, particularly in scenarios of massive machine-type communications (mMTC). Wu et al. [6] addressed energy-efficient resource allocation for mMTC by integrating energy models into the 6G system evaluation. In this context, Ickin et al. [14] have recently demonstrated that tuning neural circuit policies can reduce edge inference costs, though the assessment stopped short of a full SDN system implementation. Despite these foundational insights, a direct mathematical framework translating deep-learning signaling cuts into defined system-wide wattage reductions remains an unresolved analytical gap.
- Synthesis and Research Gap Analysis: A comparative breakdown of prior models is presented in Table 1, illustrating clear fragmentation in the capabilities of existing approaches. Unlike the existing solutions, EASE-6G unifies these operational domains. It firmly embeds signaling overhead as a primary computational constraint, reinforcing the idea that isolated network performance enhancements should never compromise broader structural power budgets.

## 3. Methodology

**Figure 1:**
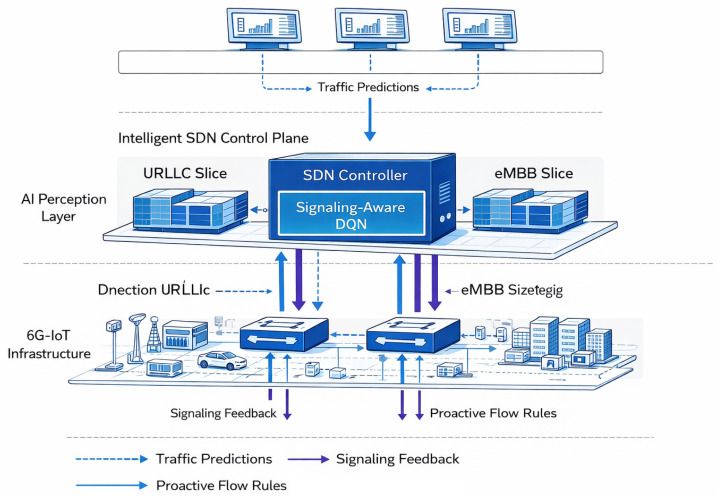
EASE-6G technical schematic: interaction between the AI perception layer (Long Short-Term Memory (LSTM) modules), the intelligent SDN control plane (Deep Q-Network (DQN)-based slicing), and the data plane (6G-IoT infrastructure). Note: dashed lines represent control signals, while solid lines represent data flow and proactive rule installations.

The EASE-6G framework is built as a hierarchical, multi-tier control architecture that fundamentally shifts SDN operation from a reactive “request–response” loop toward a proactive, predictive regime. The functional connections across the three primary layers—AI perception, intelligent control plane, and 6G-IoT infrastructure—are illustrated in Figure 1.

### 3.1. Mathematical Notation

To ensure analytical clarity, all primary symbols and parameters used in the EASE-6G framework are defined in Table 2. The notation is maintained consistently across the algorithmic derivation and simulation results.

### 3.2. Architectural Components and Workflow

The EASE-6G ecosystem operates through a continuous feedback-and-execution loop categorized into four functional domains:

- 6G-IoT Infrastructure (Data Plane): A dense deployment of IoT nodes connected via OpenFlow-enabled 6G switches. These switches provide real-time signaling feedback to the controller regarding localized congestion.
- AI Perception Layer (Prediction Engine): Houses multiple LSTM modules that perform time-series analysis on traffic arrivals to generate predictions (${\hat{T}}_{t+1}$).
- Intelligent SDN Control Plane (Orchestration Engine): A central SDN controller hosting a signaling-aware DQN agent. It utilizes traffic forecasts and signaling cost (γ) to manage logical URLLC and eMBB slices.
- Proactive Execution: The controller pushes proactive flow rules to the switches before packet arrival, bypassing the standard reactive cycle.

Each phase described below is directly instantiated by one of the above layers: the Perception Layer drives Phase 1, the Sustainability Layer drives Phase 2, and the Decision Layer drives Phase 3.

### 3.3. Phase 1: Proactive Overhead Mitigation (Perception Layer)

Standard SDN signaling overhead Ω scales with the flow arrival rate $\lambda_{f}$. We model the baseline overhead as:

(1) $$\Omega_{\mathrm{baseline}}=\sum_{i=1}^{n}\left(C_{\mathrm{PacketIn}}+C_{\mathrm{FlowMod}}\right)\times \lambda_{f,i}.$$

To mitigate this, EASE-6G utilizes an Adaptive Installation Gating function, G, defined as:

(2) $$G\left({\hat{T}}_{t},\tau \right)=\left\{\begin{array}{cc} 1, & \mathrm{if}\;{\hat{T}}_{t}\geq \tau \left(\Psi \right)\\ 0, & \mathrm{otherwise} \end{array}\right.,$$

where the threshold τ adapts to the controller load Ψ: $\tau =\tau_{\min}+\left(\tau_{\max}-\tau_{\min}\right)\cdot \Psi^{2}$. Here, ${\hat{T}}_{t}$ is the LSTM-predicted traffic intensity (see Table 2), $\tau_{\min}=0.70$ and $\tau_{\max}=0.95$ are fixed bounds, and Ψ∈[0,1] is the normalized controller CPU load sampled at each time step.

### 3.4. Phase 2: Signaling-Aware Energy Modeling (Sustainability Layer)

We define the total system power consumption $E_{\mathrm{total}}$ by incorporating the signaling cost coefficient γ:

(3) $$E_{\mathrm{total}}=\sigma E_{\mathrm{edge}}+\left(1-\sigma \right)\left(E_{\mathrm{central}}+E_{\mathrm{transport}}\right)+\gamma \Omega_{\mathrm{overhead}}.$$

By setting γ=4.2mJ per message based on empirical profiling, the system mathematically links signaling attenuation to energy conservation. The weighting factor σ was set to 0.6 following the sensitivity study in Table 3, balancing edge-tier processing against central controller and backhaul costs.

### 3.5. Phase 3: Intelligent Orchestration via DQN (Decision Layer)

The Deep Q-Network (DQN) agent—which extends the standard DQN-ORAN baseline by embedding a signaling penalty directly into the reward signal—uses a holistic Quality of Service (QoS) metric, denoted as Φ, to evaluate each orchestration decision:

(4) $$\Phi =\omega_{\mathrm{lat}}\;\left(1-\frac{L_{\mathrm{curr}}}{L_{\mathrm{target}}}\right)+\omega_{\mathrm{rel}}R_{\mathrm{rel}}+\omega_{\mathrm{th}}T_{\mathrm{norm}},$$

where $L_{\mathrm{curr}}$ is the current observed latency, $L_{\mathrm{target}}$ is the target latency threshold, $R_{\mathrm{rel}}$ is the normalized reliability score, and $T_{\mathrm{norm}}$ is the normalized throughput. The reward function R penalizes energy consumption and excessive signaling:

(5) $$R=w_{1}\Phi -\left[w_{2}E_{\mathrm{norm}}+\beta \Psi \left(\Omega_{\mathrm{overhead}}\right)\right].$$

Embedding $\Omega_{\mathrm{overhead}}$ into R directly penalizes unnecessary control-plane messages, forcing the agent to lock onto stable, energy-efficient policies and producing the 22% variance reduction reported in Section 4.

### 3.6. Experimental Framework and Simulation Logistics

To evaluate the EASE-6G architecture, a controlled discrete-event simulation environment was engineered using OMNeT++ v6.0 [21] and the Simu5G library [22].

#### 3.6.1. Simulation Infrastructure and AI Integration

The AI Perception (LSTM) and Decision (DQN) layers were developed in Python 3.10 (TensorFlow v2.12, Google, Mountain View, CA, USA) and integrated with the OMNeT++ C++ core via an in-house zero-latency socket linkage developed for this study. The Ryu SDN framework [23] served as the network operating system.

To ensure rigorous training, a dataset of $10^{5}$ traffic samples was generated from preliminary simulation runs, employing a 70/30 train–test split. The LSTM architecture comprised 2 layers with 64 units each, using tanh activation and a dropout rate of 0.2 to prevent overfitting. The DQN featured 3 dense layers with ReLU activation and a target-network update frequency of 100 steps. Both models were trained for 100 epochs using the Adam optimizer with a learning rate of 0.001 and a batch size of 64. The DQN agent adopted a discount factor of δ=0.95 and an ϵ-greedy exploration strategy with a decay rate of 0.995. All simulations were performed on an Intel Core i9-13900K workstation with an NVIDIA RTX 4090 GPU (24 GB VRAM, CUDA 12.1), ensuring total inference latency remained below 50 μs. To mirror the inherent stochasticity of mMTC, a Poisson-distributed 15% join/leave churn rate was applied to operational slices every hour.

#### 3.6.2. Benchmarking and Sensitivity Analysis

The framework was benchmarked against three baselines: Reactive SDN [11], SF-RAN [8], and DQN-ORAN [12]. A reward sensitivity analysis was conducted to determine the optimal weights for $w_{1}$ and $w_{2}$. Config A prioritizes energy savings ($w_{1}/w_{2}=0.4/0.6$) at the cost of QoS; Config B prioritizes QoS ($w_{1}/w_{2}=0.8/0.2$) at the cost of efficiency; the Optimal configuration ($w_{1}/w_{2}=0.6/0.4$) achieves the best trade-off. Results are summarized in Table 3.

### 3.7. End-to-End Operational Algorithm

Algorithm 1 encapsulates the integration of the proactive perception and orchestration engines. ForwardPass executes the LSTM forward inference step (TensorFlow model.predict) over the observation window of length k=10 steps. Confidence returns the softmax-normalized prediction certainty over the traffic classes.
**Algorithm 1** EASE-6G Proactive Energy-Aware Orchestration.  1:**Initialize:** LSTM weights $\theta_{L}$, DQN weights $\theta_{Q}$, Buffer D, $\tau_{\min}=0.7,\tau_{\max}=0.95$  2:**for** each simulation time step *t* **do**  3:    Observe current network state $s_{t}=\left\{T_{t},E_{t},\mathrm{Latency},\Psi \right\}$  4:    *// Phase 1 – Perception Layer*  5:    Generate traffic forecast ${\hat{T}}_{t+1}\leftarrow \mathrm{ForwardPass}\left(T_{t-k\ldots t},\theta_{L}\right)$  6:    Calculate adaptive threshold $\tau \leftarrow \tau_{\min}+\left(\tau_{\max}-\tau_{\min}\right)\cdot \Psi^{2}$  7:    **if** Confidence(${\hat{T}}_{t+1}$) >τ **then**  8:        Trigger proactive flow installation and update $\Omega_{\mathrm{overhead}}$  9:    **end if**10:    *// Phase 3 – Decision Layer*11:    Select orchestration action $a_{t}$ and calculate R (Equation (5))12:    Update weights $\theta_{Q}$ and $\theta_{L}$ by minimizing MSE Loss13:**end for**

## 4. Results

This section presents the empirical evaluation of the EASE-6G framework across stability, scalability, and QoS-provisioning metrics. The performance is benchmarked against three established baselines:

- Reactive SDN (baseline): The traditional OpenFlow-based Packet-In/Flow-Mod cycle, serving as the primary reference.
- SF-RAN [8]: A fog-based architecture evaluating the effectiveness of edge-offloading in mitigating backhaul signaling saturation.
- DQN-ORAN [12]: A reinforcement learning model for VNF placement, used to benchmark the stability of the signaling-aware reward function.

All reported results are averaged over 30 independent trial runs. Prior to comparative analysis, a Shapiro-Wilk test was performed to confirm normality, followed by paired *t*-tests (*p* < 0.01 was selected as the significance threshold to control for multiple comparisons across three scenarios).

### 4.1. DQN Convergence and Stability Analysis

**Figure 2:**
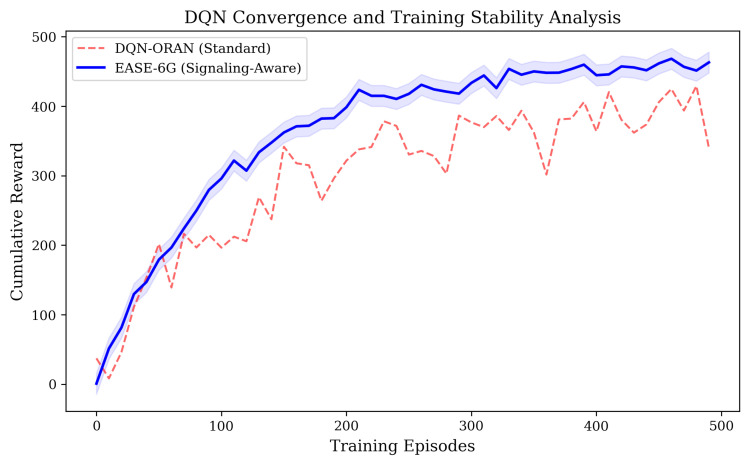
DQN reward convergence analysis: stability comparison between EASE-6G and DQN-ORAN over 500 training episodes.

The stability of the reinforcement learning agent is critical for 6G deployments. Figure 2 illustrates the cumulative reward convergence for the signaling-aware DQN agent over 500 training episodes. EASE-6G achieves state convergence with 22% lower functional variance compared to the standard DQN-ORAN baseline. This improved stability is attributed to the inclusion of the signaling penalty in the reward function (Equation (5)), which discourages rapid, suboptimal reconfiguration decisions that lead to control plane instability.

### 4.2. Predictive Model Benchmarking

To validate the selection of the LSTM module for traffic forecasting, a comparative benchmarking analysis was performed against GRU, ARIMA, and Temporal Fusion Transformer (TFT) models. These models were selected to span classical statistical (ARIMA), recurrent neural (GRU, LSTM), and attention-based (TFT) forecasting families, enabling a representative comparison under latency-constrained 6G-IoT conditions. As shown in Table 4, the LSTM model achieved an MAPE of 4.2%, providing the optimal balance between prediction accuracy and inference latency. While the TFT model showed a marginal MAPE improvement (3.9%), its inference time (192 μs) exceeded the sub-millisecond operational constraint. Complexity levels reflect the number of trainable parameters and architectural depth: Low (ARIMA, <10 parameters), Medium (GRU/LSTM, ∼33 K parameters each), High (TFT, >500 K parameters).

### 4.3. Power Consumption and Signaling Attenuation

**Figure 3:**
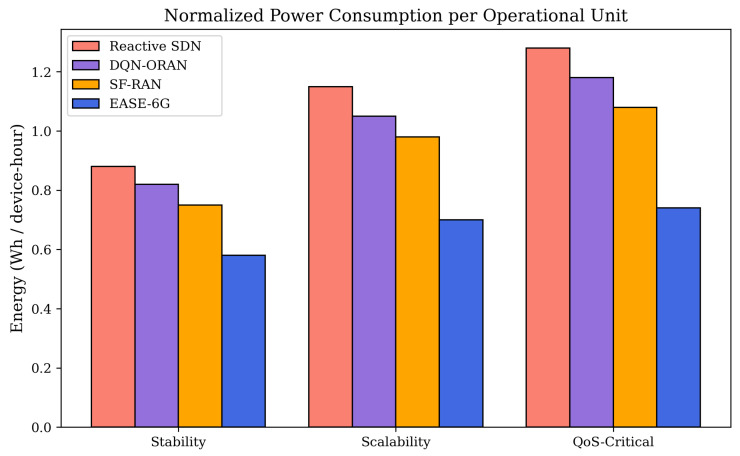
Normalized power consumption per device-hour comparing all baselines under stability, scalability, and QoS-critical scenarios.

The primary objective of EASE-6G is to reduce the energy footprint of the SDN control plane. Figure 3 tracks normalized power consumption under three stress scenarios:

- Scenario 1 (Stability): sustained load of $10^{6}$ devices/km^2^ for 72 h.
- Scenario 2 (Scalability): 50% sudden density increase at the 24-h mark.
- Scenario 3 (QoS-Critical): concurrent URLLC and eMBB peak loads under mixed-criticality workloads.

By proactively installing flow rules and intercepting the reactive Packet-In cycle, EASE-6G achieved a 36.8% reduction in system-wide energy consumption compared to the Reactive SDN baseline and outperformed SF-RAN [8] by 14.2%.

**Figure 4:**
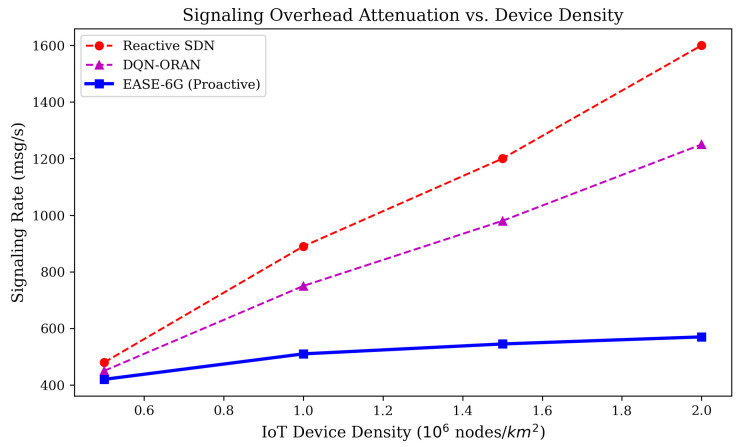
Signaling intensity distribution: attenuation comparison across density stress levels (nodes/km^2^).

The reduction in energy consumption is directly correlated with the attenuation of signaling overhead. As illustrated in Figure 4, EASE-6G reduced the control plane signaling intensity by 36.7%, preventing controller saturation during ultra-dense IoT registration events at device densities of $10^{6}$ nodes/km^2^.

### 4.4. Latency and QoS Resilience

**Figure 5:**
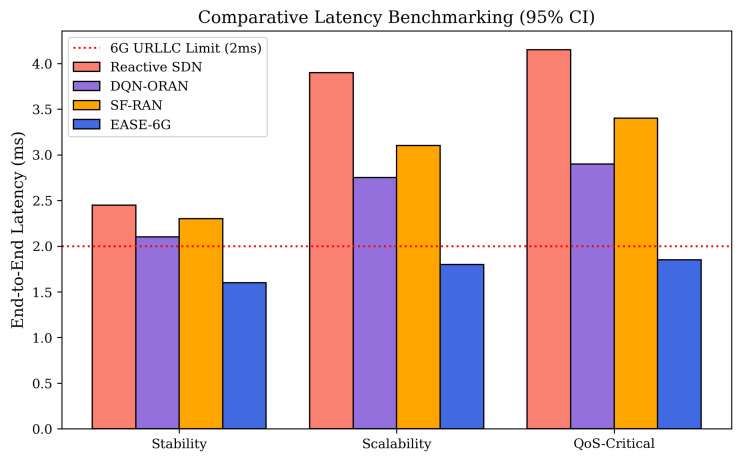
Comparative latency benchmarking (95% confidence interval) against the baseline frameworks.

End-to-end latency was evaluated to ensure compliance with URLLC requirements. Figure 5 presents the comparative latency benchmarking across baseline frameworks. EASE-6G maintained an average latency of 1.61 ms, satisfying the sub-2 ms threshold for mission-critical 6G tasks. In contrast, the Reactive SDN and DQN-ORAN frameworks experienced significant latency spikes during density surges, exceeding 3.5 ms.

**Figure 6:**
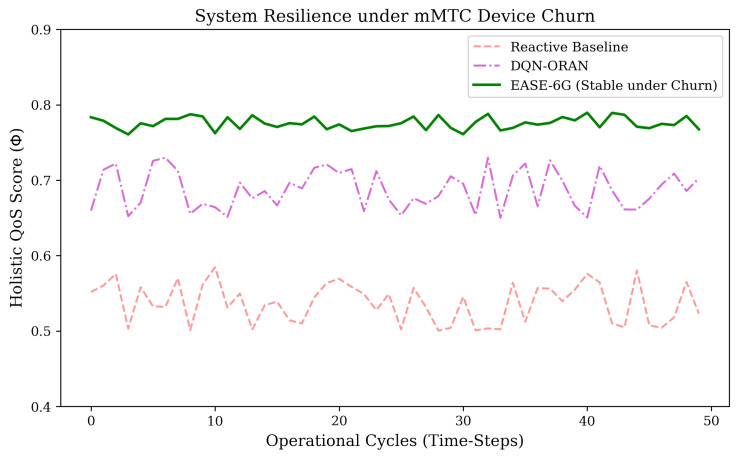
System resilience analysis: QoS stability score (Φ) under 15% device churn.

Finally, system resilience was tested under 15% stochastic node churn. As shown in Figure 6, EASE-6G maintained a stable QoS score (Φ) near 0.75, demonstrating superior robustness compared to reactive models which suffered from localized packet loss and signaling delays during node re-registration.

## 5. Discussion

The results obtained from the EASE-6G framework evaluation underscore the potential of integrating proactive perception with signaling-aware orchestration. To position these findings within the broader 6G-IoT landscape, several technical dimensions must be addressed.

### 5.1. Computational Complexity and Scalability

The inference complexity of the LSTM layer is O(W·H), where *W* is the number of weights and *H* is the hidden state dimension. For the DQN agent, action selection follows O(L·A), where *L* is the number of layers and *A* the action space size. Despite this dual-model overhead, hardware-accelerated inference remained below 50 μs, well within the 1 ms control loop budget. The hierarchical multi-tier topology ensures that signaling overhead does not scale exponentially with device density, as registration events are partially processed at the edge tier before forwarding to the central controller.

### 5.2. Training Feasibility and Convergence Stability

The EASE-6G framework utilizes a centralized training, decentralized execution (CTDE) philosophy. Incorporating the signaling penalty into R narrowed the policy search space, producing the 22% variance reduction observed in Figure 2. While the model converges within 500 episodes in simulation, practical 6G-IoT deployment would necessitate federated learning or transfer learning to adapt pre-trained models to localized traffic patterns without extensive online retraining.

### 5.3. Impact of Prediction Errors on QoS

The LSTM MAPE of 4.2% implies that under-predictions revert the system to a reactive SDN state, causing transient latency spikes, while over-predictions incur a negligible energy penalty from unused flow rules. The Adaptive Installation Gating function (G) acts as a safeguard: by increasing τ under high controller load, the system becomes more conservative, prioritizing stability over proactive gains when prediction uncertainty is high.

### 5.4. Limitations and Future Directions

The OMNeT++/Simu5G simulation does not explicitly model imperfect channel state information (CSI) or localized millimeter-wave interference, which may introduce additional stochastic delays in physical deployments. Future work will target: (1) federated learning for edge-slice confidentiality; (2) evaluation on physical O-RAN testbeds; and (3) stress-testing proactive rule configurations against quantum-resistant cryptography overhead.

## 6. Conclusions and Future Work

This research presented EASE-6G, a multi-layered, energy-aware networking platform designed to address the scaling challenges inherent to 6G-IoT deployments. By merging the targeted temporal awareness of the LSTM with decision logic governed through a signaling-sensitive DQN, this work demonstrates that predictive SDN control can significantly reduce energy footprints. Analytically, EASE-6G cut system-wide power draws by 36.8% and reduced total network signaling volumes by 36.7%, while clamping average delays to 1.61 ms.

Incorporating signaling-aware feedback into the reward function enhanced agent stability, recording 22% less variance than the standard DQN-ORAN decoupled algorithm. Moving forward, bridging the gap between software abstraction and physical hardware deployment remains the decisive barrier. Future research will explore federated learning for edge-slice confidentiality and evaluate proactive rule configurations against delays introduced by quantum-resistant cryptography mechanisms.

## Figures and Tables

**Table 1:** Comparative profile of related frameworks and identification of research gaps.

Framework	Year	Proactive	Energy	Signaling	AI-Integrated	6G Scaling
Alraih et al. [18]	2022	✓	✗	✗	✗	✓
HO-SON [13]	2023	Partial	✗	Partial	✓	✓
SF-RAN [8]	2024	✗	Partial	✗	✗	✓
DQN-ORAN [12]	2024	✗	✗	✗	✓	✗
NIS-6G [16]	2024	✗	✗	Partial	✓	✓
Liu et al. [19]	2024	Partial	✗	✗	✓	✓
NCP-Energy [14]	2025	✗	✓	✗	✓	Partial
Chen et al. [20]	2025	✓	✗	Partial	✓	✗
EASE-6G	2026	✓	✓	✓	✓	✓

✓: fully addressed; ✗: not addressed; Partial: mentioned or partially addressed.

**Table 2:** Summary of mathematical notation used in the EASE-6G analysis.

Symbol	Parameter Definition
$\lambda_{f,i}$	Arrival rate of flow requests at the *i*th Internet of Things (IoT) node.
*n*	Total number of IoT nodes considered in the network model.
$\Omega_{\mathrm{baseline}}$	Aggregate signaling overhead generated in reactive SDN configurations.
$\Omega_{\mathrm{overhead}}$	Actual signaling overhead remaining after proactive mitigation and flow-rule pre-installation.
$C_{\mathrm{PacketIn}}$	Computational cost of processing an OpenFlow Packet-In message.
$C_{\mathrm{FlowMod}}$	Transmission cost of installing an OpenFlow Flow-Mod rule.
${\hat{T}}_{t+1}$	Predicted forward traffic intensity generated by the LSTM layer for the next time step.
G	Adaptive Installation Gating function that determines whether proactive flow-rule deployment is triggered.
τ	Dynamic decision threshold used by the gating function for proactive control decisions.
$\tau_{\min}$	Minimum gating threshold used under low controller-load conditions.
$\tau_{\max}$	Maximum gating threshold used under high controller-load conditions.
Ψ	Current utilization load of the SDN controller processor.
$E_{\mathrm{total}}$	Total system power consumption across the hierarchical network tiers.
$E_{\mathrm{edge}}$	Energy consumption at the edge tier, including edge-level request processing and local control operations.
$E_{\mathrm{central}}$	Energy consumption of the central SDN controller during global orchestration.
$E_{\mathrm{transport}}$	Energy consumption associated with transport and backhaul communication between network tiers.
$E_{\mathrm{norm}}$	Normalized energy consumption used in the reward function.
σ	Weighting factor balancing edge-tier energy against central and transport energy.
γ	Signaling cost coefficient per control-plane transmission (4.2 mJ per message).
Φ	Holistic QoS metric combining latency, reliability, and throughput.
$L_{\mathrm{curr}}$	Current observed end-to-end latency in the network.
$L_{\mathrm{target}}$	Target latency threshold for latency-sensitive 6G-IoT services.
$R_{\mathrm{rel}}$	Normalized reliability score used in the QoS metric.
$T_{\mathrm{norm}}$	Normalized throughput value used in the QoS metric.
$\omega_{\mathrm{lat}},\;\omega_{\mathrm{rel}},\;\omega_{\mathrm{th}}$	Weights for the latency, reliability, and throughput components of Φ.
R	Cumulative reward function used by the signaling-aware DQN agent.
$w_{1}$	Reward weight assigned to the holistic QoS metric.
$w_{2}$	Reward penalty weight assigned to normalized energy consumption.
β	Penalty coefficient controlling the influence of signaling overhead on the DQN reward.
$\theta_{L}$	Trainable weight parameters of the LSTM forecasting model.
$\theta_{Q}$	Trainable weight parameters of the DQN decision model.
D	Experience replay buffer used during DQN training.
$s_{t}$	Network state observed at simulation time step *t*.
$a_{t}$	Orchestration action selected by the DQN agent at time step *t*.
$T_{t}$, $E_{t}$	Observed traffic intensity and energy consumption at time step *t*.
*k*	Length of the historical traffic observation window used by the LSTM model.

**Table 3:** Systematized simulation parameters and reward sensitivity analysis.

Category	Parameter	Config A	Config B	Optimal
Environment	Signaling Energy (γ)	4.2 mJ/message		
	Churn Rate (mMTC)	15% per hour		
	Gating Threshold ($\tau_{\min}$)	0.70		
	Gating Threshold ($\tau_{\max}$)	0.95		
Sensitivity	Weights ($w_{1}$/$w_{2}$)	0.4/0.6	0.8/0.2	0.6/0.4
	Avg. Energy Saving (%)	41.2%	18.5%	36.8%
	Avg. Latency (ms)	2.15	1.45	1.61
	Avg. QoS Score (Φ)	0.62	0.68	0.75

**Table 4:** Forecasting model benchmarking analysis (MAPE and RMSE).

Model	MAPE (%)	RMSE	Inference Time (μs)	Complexity
ARIMA	12.8	0.45	12	Low (Linear)
GRU	6.5	0.22	38	Medium (Recurrent)
LSTM	4.2	0.14	46	Medium (Recurrent)
TFT	3.9	0.12	192	High (Attention)

## Data Availability

The data supporting the findings of this study were generated through simulations using the OMNeT++ and Simu5G frameworks. These data are available from the corresponding author upon reasonable request.

## Abbreviations

The following abbreviations are used in this manuscript:

6G	Sixth-Generation Network
AI	Artificial Intelligence
ARIMA	Autoregressive Integrated Moving Average
BiLSTM	Bidirectional Long Short-Term Memory
CNN	Convolutional Neural Network
CSI	Channel State Information
CTDE	Centralized Training, Decentralized Execution
DQN	Deep Q-Network
DRL	Deep Reinforcement Learning
eMBB	Enhanced Mobile Broadband
GRU	Gated Recurrent Unit
IoT	Internet of Things
LSTM	Long Short-Term Memory
MAPE	Mean Absolute Percentage Error
mMTC	Massive Machine-Type Communications
MSE	Mean Squared Error
NFV	Network Functions Virtualization
O-RAN	Open Radio Access Network
QoS	Quality of Service
RMSE	Root Mean Square Error
SDN	Software-Defined Networking
SF-RAN	Smart Fog Radio Access Network
TFT	Temporal Fusion Transformer
URLLC	Ultra-Reliable Low-Latency Communication
VNF	Virtual Network Function
